# The Vitamin D Receptor Agonist BXL-01-0029 as a Potential New Pharmacological Tool for the Treatment of Inflammatory Myopathies

**DOI:** 10.1371/journal.pone.0077745

**Published:** 2013-10-30

**Authors:** Luigi Di Luigi, Mariangela Sottili, Cristina Antinozzi, Gabriella Barbara Vannelli, Francesco Romanelli, Valeria Riccieri, Guido Valesini, Andrea Lenzi, Clara Crescioli

**Affiliations:** 1 Department of Movement, Human and Health Sciences, University of Rome “Foro Italico”, Rome, Italy; 2 Excellence Center for Research, Transfer and High Education De Novo Therapies (DENOthe), University of Florence, Florence, Italy; 3 Department of Experimental and Clinical Medicine, University of Florence, Florence, Italy; 4 Department of Experimental Medicine, Sapienza University of Rome, Rome, Italy; 5 Department of Internal Medicine and Clinical Specialities, Sapienza University of Rome, Rome, Italy; Roswell Park Cancer Institute, United States of America

## Abstract

**Objective:**

This study aims to investigate *in vitro* the effect of the VDR agonist BXL-01-0029 onto IFNγ/TNFα-induced CXCL10 secretion by human skeletal muscle cells compared to elocalcitol (VDR agonist), methylprednisolone, methotrexate, cyclosporin A, infliximab and leflunomide; to assess *in vivo* circulating CXCL10 level in subjects at time of diagnosis with IMs, before therapy, together with TNFα, IFNγ, IL-8, IL-6, MCP-1, MIP-1β and IL-10, vs. healthy subjects.

**Methods:**

Human fetal skeletal muscle cells were used for *in vitro* studies; ELISA and Bio-Plex were used to measure cell supernatant and IC_50_ determination or serum cytokines; Western blot and Bio-Plex were for cell signaling analysis.

**Results:**

BXL-01-0029 decreased with the highest potency IFNγ/TNFα-induced CXCL10 protein secretion and targeted cell signaling downstream of TNFα in human skeletal muscle cells; CXCL10 level was the highest in sera of subjects diagnosed with IMs before therapy and the only one significantly different vs. healthy controls.

**Conclusions:**

Our *in vitro* and *in vivo* data, while confirm the relevance of CXCL10 in IMs, suggested BXL-01-0029 as a novel pharmacological tool for IM treatment, hypothetically to be used in combination with the current immunosuppressants to minimize side effects.

## Introduction

The idiopathic inflammatory myopathies (IMs) cover a heterogeneous group of systemic autoimmune diseases which share chronic inflammation and infiltration by inflammatory cells in skeletal muscles, despite the distinct immune effector mechanisms underlying the specific disease subtypes - mainly dermatomyositis, polymyositis, inclusion body myositis, necrotizing autoimmune myositis or myositis associated with systemic disorders. Macrophages, dendritic cells (DCs) and T cells with T helper (Th) 1 immune reaction predominance are, indeed, prominently present in muscles of the different IM types [1], [2].

Local accumulation of T cells and macrophages likely contribute to the deposition of immune complexes within skeletal muscles [3], [4] by releasing functional molecules, such as cytokines and chemokines.

Nowadays, the concept that skeletal muscle cells behave as immunoactive counterpart dialoguing with immune system during inflammation has been widely accepted. Muscle cells actively participate to inflammation by promoting cytokine-mediated T cell invasion [5]–[7]. Among the wide range of proinflammatory cytokines highly involved in IMs [1], [5], interferon (IFN)γ and tumor necrosis factor (TNF)α, both with strong Th1 association, have been documented to be upregulated in muscle tissue and in serum of IM patients [5]–[8]. The IFNγ inducible 10 kDa protein, CXCL10/IP-10, a powerful chemokine known to initiate and amplify Th1 cell infiltration in several tissue/cell types during inflammation [9]–[13], seems to play a pivotal role in muscles of subjects with IMs during the early inflammatory signals, as well [1], [14]–[16]. At the onset of several Th1-mediated (auto)immune diseases, CXCL10 can alter the Th1/Th2 balance [14], driving early T cell response towards Th1 type immune polarization and dominance. Tissue/cell accumulation of CXCL10 is thought to trigger/perpetuate a self-promoting inflammatory loop throughout the interaction with its specific receptor CXCR3 on Th1 cells [13], [17]. We’have recently reported that human skeletal muscle cells (Hfsmc), an *in vitro* cell system fully chararcterized and optimized, secreted CXCL10 when challenged by inflammatory stimuli, likely throughout a TNFα-driven mechanism [10]. Current therapies for IMs, such as corticosteroids and second-line immunosuppressants - aimed to reduce the side effects of corticosteroid - are designed to target immune cells. The observation that nearly 25% of the patients do not respond to those drugs and are left with disability has been driving the attention onto the need for new pharmacological tool(s), hopefully targeting also the muscular component [18]. Some current immunosuppressants, such as methylprednisolone (MeP), methotrexate (MTX), cyclosporin A (CsA) and infliximab have been shown to exert a little effect onto CXCL10 secretion by human skeletal muscle cells (Hfsmc) [10].

Vitamin D receptor (VDR) agonists have emerged to exert pleiotropic activities in (auto)immune regulation [19]. Remarkably, they attenuated Th1-mediated inflammation during either auto- or alloresponse by targeting both immune and resident cells [9], [20], [21], thus becoming suitable candidates as novel immunosuppressants in autoimmune diseases and transplantation [22], [23]. In particular, we have previously shown that two VDR agonists, BXL-01-0029 and elocalcitol, both retaining vitamin D biologic activity - with less or without hypercalcemic side effects, respectively - significantly counteracted CXCL10 secretion by human thyrocytes, cardiomyocytes and renal tubular cells [9],[20],[21]. In particular, BXL-01-0029, a prodrug of BXL-2198 (also known as BXL-219 and Ro 26–2198), has been shown to be active onto Th1-mediated inflammatory processes in animal models of autoimmune type 1 diabetes [24] and colonic carcinogenesis [25]; elocalcitol, proposed for benign prostate hyperplasia treatment, retains anti-inflammatory properties as documented in experimental model of autoimmune prostatitis [26], [27].

Herein, we aimed to investigate whether and how BXL-01-0029, in comparison with elocalcitol, MeP, MTX, CsA, infliximab and leflunomide (LEF) [28], [29], exert any effect onto IFNγ- and TNFα-induced CXCL10 protein secretion by Hfsmc.

Furthermore, we aimed to assess CXCL10 level in serum of subjects at the time of diagnosis with different subtypes of IMs and before therapy initiation, together with other cytokines, such as TNFα, IFNγ, interleukin (IL)-8, IL-6, monocyte chemoattractant protein-1 (MCP-1), macrophage inflammatory protein 1 (MIP-1)β, categorized as Th1 type and all involved in muscular inflammation [30], [31], and IL-10, classified as Th2 type; sex- and age-matched healthy subjects were used as controls.

## Materials and Methods

### Chemicals

DMEM/Ham’s F-12 medium (1∶1) with and without phenol red, phosphate buffered saline (PBS) Ca^2+^/Mg^2+^-free, bovine serum albumin (BSA) fraction V, glutamine, antibiotics, collagenase type IV, NaOH, Bradford reagent, 4′,6-Diamidino-2-phenylindole (DAPI), all reagents for western blot, signal transducer and activator of transcription-1 (Stat-1) inhibitor fludarabine, phosphatidylinositol 3-kinase (PI3K) inhibitor LY294002, p38 mitogen-activated protein kinase (MAPK) inhibitor SB 203580, extracellular signal-regulated kinase (ERK) inhibitor U0126, C-Jun NH2-terminal kinase (JNK) inhibitor (SP600125), MeP, CsA, MTX and LEF were from Sigma Aldrich (St. Louis, MO, USA). Fetal calf serum was from HyClone (Logan, UT). 2-mercaptoethanol were from Life Technologies, Inc. Laboratories (Grand Island, NY). INFγ, TNFα, IL-12, IL-18, IL-2, IL-4 and ELISA kits for human CXCL10 were from R&D Systems, Inc. (Minneapolis, MN, USA).

BXL-01-0029 and elocalcitol were from Bioxell (Milan, Italy). Nuclear factor-kB (NF-kB) inhibitor BAY 11–7082 was from Vinci Biochem S.r.l. (Vinci, Italy). Chimeric monoclonal antibody infliximab (Remicade) was from Centocor B.V. (Leiden, The Netherlands).

Antibodies (Abs) for western blot and immunocytochemical analysis: rabbit polyclonal primary anti-phospho Tyr701 Stat-1 (p-Stat-1), mouse monoclonal primary anti-phospho Ser536 Nuclear factor-kB (p-NF-kB), rabbit polyclonal primary anti-Stat-1 were from Cell Signaling (Danvers, MA, USA); rabbit polyclonal primary anti-phospho Thr183/Tyr185 JNK (p-JNK), rabbit polyclonal primary anti-JNK/SAPK1, peroxidase secondary Abs, all reagents for SDS-PAGE were from Millipore (Billerica, MA, USA); mouse monoclonal primary anti-β actin, rabbit polyclonal anti-human primary anti-NF-kB p65 (C-20) were from Santa Cruz Biotechnology (Santa Cruz, CA, USA); mouse monoclonal anti-human leucocyte antigen (HLA) class I, Cy3-labelled secondary antibody were from Sigma Aldrich (St. Louis, MO, USA); mouse monoclonal anti-HLA class II-FITC conjugated was from BD Biosciences (San Jose, California, USA). Plastic ware for cell cultures and disposable filtration units for growth media preparation were purchased from Corning (Milan, Italy).

### Cell Cultures

Cells were obtained as previously described [10]. Hfsmc were isolated from 11 fetal skeletal male muscles (four upper and seven lower limbs) obtained after voluntary abortion (10–12 weeks of gestation). Legal abortions were performed in authorized hospitals and written consent was given by the patients for their human fetal tissue to be stored and used for research. The use of human fetal tissue for research purposes was approved by the Committee for investigation in humans of the Azienda Ospedaliero-Universitaria Careggi, Florence, Italy (protocol n° 6783-04). All samples have been handled in the same way and maintained in ice-cold PBS until processed for culture preparation as described elsewhere [10]. Hfsmc express skeletal muscle specific linage markers, either proteins and genes - tropomyosin, myosin 1β, sarcomeric actin, myostatin, heavy chain myosin, myogenin, MyoD, Pax 3 and 7 - and display a high degree of myogenic purity and reproducibility; they fuse in myotubes under appropriate conditions and spontaneously retain the functional competence of phenotypically mature skeletal muscular cells together with proliferation ability - albeit with a limited lifespan - therefore representing an unique tool for basic research [10]. Confluent cell cultures were split into a 1∶2–1∶4 ratio using EDTA-trypsin solution (0.2–0.5%), and used from 5^th^ to 12^th^ passage (5p–12p).

### Subjects

We studied 20 Caucasian patients (13 females and 7 males). Approval by the Local Ethics Committee, Azienda Policlinico Umberto I Rome Italy, accordance with the principles outlines in the Declaration of Helsinki, was obtained. Written consents were obtained. The group included: patients with dermatomyositis (n = 8), polymyositis (n = 7), polymyositis associated with other connective tissue diseases (n = 3, one with rheumatoid arthritis, one with systemic sclerosis and one with Sjogren’s syndrome), inclusion body myositis (n = 2).

The mean age of patients was 56.4 years (range: 38–82). Caucasian healthy blood donors (n = 20) sex- and age-matched without any sign of myositis, arthritis, ongoing inflammatory or autoimmune conditions, were used as controls in accordance with Local Ethical Committee approval. Written informed consents were obtained.

### Serum Samples

Sera from patients were obtained from the blood samples at time of muscular biopsy for IM diagnosis, before pharmacological treatment with immunosuppressants; control sera were obtained from blood of healthy anonymous donors. All blood samples were collected from peripheral vein and serum was obtained by centrifugation (3000 rpm for 10 min at 4°C); aliquots were stored at −80°C until analyzed.

### Cytokine Secretion Assays

For CXCL10 secretion assays, 4000 cells/well were seeded in 96-well flat bottom plates and maintained in serum deprivation as previously described [10]. Thereafter, different stimuli were added in serum-free medium with 0.1% BSA, according to the experimental protocols; cells in serum-free medium containing 0.1% BSA and vehicle were used as control.

For dose-response experiments cells were stimulated for 24 h with IFNγ (1000 U/ml)+TNFα (10 ng/ml), with or without BXL-01-0029 and elocalcitol (10^−11^ or 10^−10^–10^−6 ^M), MTX (1×10^−7^, 2×10^−7^, 4×10^−7^, 8×10^−7^, 1.6×10^−6^ M), CsA (4×10^−8^, 8×10^−8^, 2×10^−7^, 4×10^−7^, 8×10^−7^ M), MeP (1.3×10^−7^, 3×10^−7^, 7×10^−7^,1×10^−6^, 3×10^−6^ M), infliximab (3×10^−9^, 7×10^−9^, 2×10^−8^, 3×10^−8^, 7×10^−8^, 2×10^−7^ M) or LEF (9×10^−6^, 2×10^−5^, 4×10^−5^, 9×10^−5^, 1.8×10^−4^, 3.7×10^−4^ M). The drug concentrations were selected on the basis of their near-therapeutic doses, according to their pharmacokinetics (C_max_ and area under the time concentration curves, AUC). Supernatants were harvested and kept frozen at −20°C until performing ELISA assays.

For experiments with cytokine combination Hfsmc were incubated for 24 h with IFNγ (1000 U/ml) or TNFα (10 ng/ml), alone or combined; either IFNγ (1000 U/ml) or TNFα (10 ng/ml) were combined with IL-12 (50 ng/ml), IL-18 (50 ng/ml), IL-2 (25 U/ml) and IL-4 (20 ng/ml). The doses for cytokine combination have been previously optimized and selected on the basis of the maximal evoked synergistic response, in terms of CXCL10 protein secretion [10].

For experiments with inhibitors of intracellular pathways cells were incubated for 24 h with a combination of IFNγ (1000 U/ml)+TNFα (10 ng/ml) with or without 1 h pre-treatment with fludarabine (50 µM), LY294002 (15 µM), SB 203580 (5 µM), U0126 (20 µM), BAY 11–7082 (20 µM), SP600125 (100 µM). Experiments were performed at least five time in triplicate with different cell preparations.

### ELISA

CXCL10 levels were measured in cell culture supernatants using commercially available kits, according to manufacturer’s recommendations. The sensitivity and the intra- and inter assay coefficients of variation were indicated in manufacturer’s instructions. Quality control pools of low, normal, or high concentrations for all parameters were included in each assay. The amount of CXCL10 was expressed as pg/µg of total protein amount or as percent of IFNγ-, TNFα- or IFNγ+TNFα-induced secretion, as appropriate. Protein extraction and measurement to normalize Hfsmc secretion were performed as reported elsewhere [11]. Experiments were performed at least in triplicate with at least five different cell preparations.

### Immunoblot Analysis

For protein analysis, Hfsmc, seeded and maintained in the same conditions as previously reported [10], were stimulated with TNFα (10 ng/ml) or IFNγ (1000 U/ml) for 12 minutes in presence or absence of BXL-01-0029 (10^−8^ M) in serum-free medium containing 0.1% BSA. Cells in serum-free medium with 0.1% BSA and vehicle were used as control. Protein concentration measurement was performed with Bradford Reagent.

Protein aliquots (20 µg) were processed, loaded onto 10% SDS-PAGE and transferred on PVDF membranes, according to the procedure previously described [10]. Thereafter, membranes were incubated with primary Abs appropriately diluted in Tween Tris-buffered saline (TTBS) (for anti-p-Stat-1, anti-Stat-1, anti-p-JNK, anti-JNK, anti-p-NF-kB, anti-NF-kB 1∶1000; for anti-β actin 1∶10000), followed by peroxidase-conjugated secondary IgG (1∶10000). Proteins were revealed by the enhanced chemiluminescence system (ECL plus; Millipore). Image acquisition were performed with Image Quant Las 4000 software (GE Healthcare) and densitometric analysis with Quantity One® software (Bio-Rad laboratories Inc.). Western blot analysis was performed for three/four independent experiments with different cell preparations.

### Bio-Plex Phosphoprotein Assay

After stimulation, Hfsmc were washed with ice-cold PBS, harvested and lysed in a phosphoprotein-lysis-buffer (Bio-Rad Laboratories, Inc.). After centrifugation (4500 *g*, 20 min, 4°C), the supernatant containing the phosphoprotein was collected and protein measurement performed.

Phospho-ERK1/2 were measured in triplicates using the Bio-Plex protein array system (Bio-Rad Laboratories, Inc.), according to the manufacturer’s protocol.

The system is a multiplexed particle-based flow cytometric assay that utilizes anti-phosphokinase monoclonal antibodies linked to microspheres incorporating distinct proportions of two fluorescent dyes. Experiments were performed in triplicate with different cell preparations.

### Immunocytochemistry

To evaluate class I and II HLA protein expression, 10^4^ cells were seeded on glass coverslips in growth medium, maintained in serum-free medium overnight and then treated with TNFα (10 ng/ml)+IFNγ (1000 U/ml) for 24 and 48 hours, with and without BXL-01-0029 (10^−8^ M), or with VDR agonist alone. Cells in phenol red- and serum-free medium 0.1% BSA and vehicle were used as control. Immunostaining procedure was as previously described [10]; primary Ab against class I HLA was used at 1∶5 dilution and was followed by CY3 conjugated mouse secondary Ab (1∶200); FITC-conjugated Ab against class II HLA was used at 1∶10 dilution. DAPI nucleic acid stain was used to stain nuclei (1∶10000). Slides lacking the primary Ab or stained with the corresponding nonimmune serum were processed for method specificity. Slides were examined with Zeiss Z1 microscope and Leica TCS SP2 (Leica, Milano, Italy); images were acquired at 100X magnification. For quantification: the percentage of class I or II HLA positive cells was calculated by counting the number of stained cells over the total in at least 15 separate field per slides. Experiments were performed four times with different cell preparations.

### Multiplex Cytokine Assay

Serum levels of CXCL10, TNFα, IFNγ, IL-8, IL-6, MCP-1 MIP-1β, IL-10 were measured using a magnetic bead-based multiplex assay (Bio-Plex Pro™ Human Cytokine, Chemokine, and Growth Factor Assay, Bio-Rad Laboratories, Inc.) according to the manufacturer’s protocol. A broad sensitivity range of standards (Bio-Rad Laboratories, Inc.), ranging between 1.95 and 95000 pg/ml were used to help enable the quantitation of a dynamic wide range of cytokine concentrations and provide the greatest sensitivity. Data acquisition was performed by Bio-Plex 200 System™ (Bio-Rad Laboratories, Inc.) which uses Luminex fluorescent-bead-based technology (Luminex) with a flow-based dual laser detector with real-time digital signal processing to facilitate the analysis of up to 100 different families of colour-coded polystyrene beads and allow multiple measurements of the sample ensuing in the effective quantification of cytokines. Data analysis was performed by Bio-Plex Manager™ 6.0 software (Bio-Rad Laboratories, Inc.). Serum samples were run in triplicate twice.

### Statistical Analysis

The statistical analysis was performed using GraphPad Prism 5 software (GraphPad Software, Inc., La Jolla, CA, USA) and SPSS 12.0 software package (SPSS for Windows 12.0, SPSS Inc., Chicago, IL, USA). The Kolmogorov-Smirnov test for normal distribution of the data, one-way analysis of variance (ANOVA), T-test were applied.

A P value less than 0.05 was considered significant and corrected for comparison using the Dunnett’s or Bonferroni’s post hoc test, where appropriate. The computer program ALLFIT (NIH, Bethesda, MD, USA) [32] was used for analyses of sigmoid dose-response curves to obtain estimates of IC_50s_ of BXL-01-0029, elocalcitol, MTX, CsA, MeP, infliximab and LEF. Data are expressed as mean±S.E.

## Results

### BXL-01-0029 Inhibited CXCL10 Secretion by Hfsmc with the Highest Potency

Drug pharmacologic potency was determined in Hfsmc incubated with IFNγ (1000 U/ml)+TNFα (10 ng/ml) and increasing concentrations of BXL-01-0029, elocalcitol (10^−11^ or 10^−10^–10^−6 ^M), MTX (1×10^−7^, 2×10^−7^, 4×10^−7^, 8×10^−7^, 1.6×10^−6^ M), CsA (4×10^−8^, 8×10^−8^, 2×10^−7^, 4×10^−7^, 8×10^−7^ M), MeP (1.3×10^−7^, 3×10^−7^, 7×10^−7^, 1×10^−6^, 3×10^−6 ^M), infliximab (3×10^−9^, 7×10^−9^, 2×10^−8^, 3×10^−8^, 7×10^−8^, 2×10^−7^ M) and LEF (9×10^−6^, 2×10^−5^, 4×10^−5^, 9×10^−5^, 1.8×10^−4^, 3.7×10^−4^ M).

**Figure 1:**
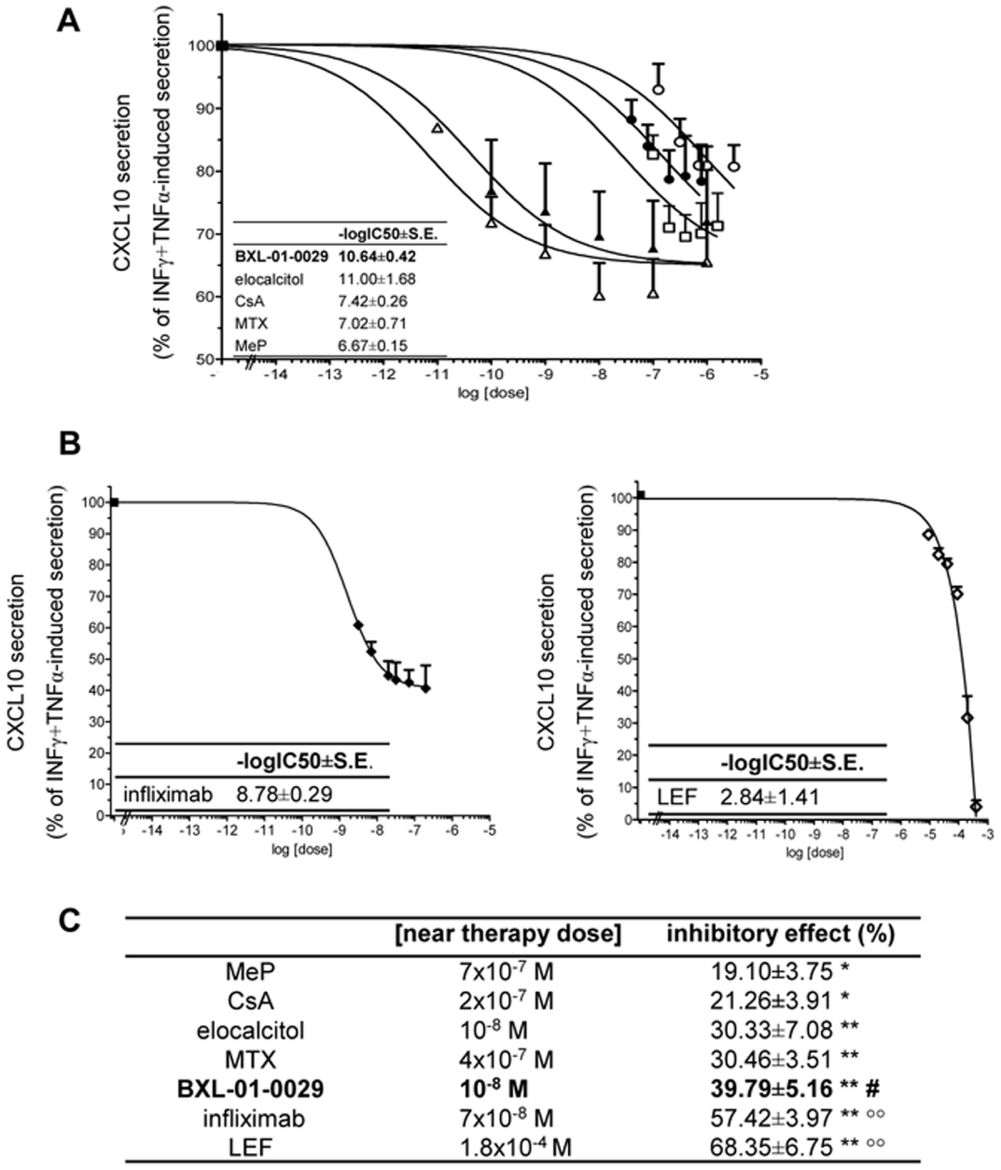
Effect of different immunosuppressors on cytokine-induced CXCL10 secretion in Hfsmc. A, BXL-01-0029 (open triangles), elocalcitol (closed triangles), MTX (open squares), CsA (closed circles) and MeP (open circles) dose-dependently inhibited IFNγ+TNFα-induced CXCL10 secretion (closed squares, taken as 100%, ) after 24 h. Simultaneous fitting of the curves showed that BXL-01-0029 and elocalcitol retained the highest potency, as shown by the IC_50s_ depicted in the inset of the figure. B, Neither infliximab (closed losangues, left) nor LEF (open losangues, right) exerted a dose-dependent inhibition of cytokine-induced CXCL10 secretion (closed squares, taken as 100%); IC_50 s_ are reported in figure insets. C, At concentrations corresponding to the near therapy doses the inhibition by BXL-01-0029, not by elocalcitol, was significantly higher than MeP-, CsA- and MTX-induced ones (*P<0.05 or **P<0.01 vs. IFNγ+TNFα-induced secretion, ^#^P<0.05 vs. all treatment except for elocacitol, °°P<0.01 vs. all other drugs). Results (mean±S.E.) are expressed as % of IFNγ+TNFα-induced CXCL10 secretion (taken as 100%) or % of inhibition. Data are obtained from five experiments with different cell preparations.

As revealed by dose-response curves (Figure 1A and 1B), all drugs significantly reduced IFNγ+TNFα-induced CXCL10 release by Hfsmc (P<0.05 or P<0.01 vs. IFNγ+TNFα-induced secretion, taken as 100%, starting from the first or the second lowest drug concentrations, Figure 1A: BXL-01-0029, elocalcitol, MTX, CsA, MeP; Figure 1B: infliximab and LEF). CXCL10 secretion was virtually absent in control cells (not shown).

In particular, infliximab and LEF (Figure 1B, left and right, respectively) did not display a dose-dependent effect similar to the other tested drugs; instead, they both appeared to be toxic onto Hfsmc (not shown). The cytotoxic effect may explain, at least in part, the highest maximal inhibition of infliximab and LEF onto CXCL10 secretion observed at fixed concentrations corresponding to the related near therapy doses (Figure 1C, P<0.01 vs. control and vs. all other drugs). The inhibition by BXL-01-0029 at the near therapy dose (39.79±5.16%) - differently from elocalcitol (30.33±7.08%) - was significantly higher (P<0.05) than that one observed with MeP (19.10±3.75%), CsA (21.26±3.91%) and MTX (30.46±3.51%).

However, both VDR agonists were the most potent as compared to the other drugs, as shown by the calculated IC_50_, that are: MeP: −log IC_50_ = 6.67±0.15; MTX: −log IC_50_ = 7.02±0.71; CsA: −log IC_50_ = 7.42±0.26; BXL-01-0029 and elocalcitol: −log IC_50_ = 10.64±0.42 and −log IC_50_ = 11.00±1.68, respectively (inset of Figure 1A); infliximab −log IC_50_ = 8.78±0.29 and LEF: −log IC_50_ = 2.84±1.41, (inset of Figure 1B, left and right). In table 1 the P values for each IC_50_ vs. the other ones are also reported; BXL-01-0029 displayed an IC_50_ value statistically significant vs. all the other tested drugs, but elocalcitol.

**Table 1:** Comparison of the calculated IC_50 s_.

	P value						
	BXL-01-0029	elocalcitol	infliximab	CsA	MTX	MeP	LEF
**BXL-01-0029**	**–**	**ns**	**0.0026**	**0.0002**	**0.0145**	**0.0070**	**<0.0001**
elocalcitol	**Ns**	–	0.0452	ns	0.0408	0.0122	0.0077
infliximab	**0.0026**	0.0452	–	ns	0.0323	0.0147	<0.0001
CsA	**0.0002**	ns	ns	–	ns	0.0067	Ns
MTX	**0.0145**	0.0408	0.0323	ns	–	Ns	0.0385
MeP	**0.0070**	0.0122	0.0147	0.0067	ns	–	0.0397
LEF	**<0.0001**	0.0077	<0.0001	ns	0.0385	0.0397	–

P values for each IC_50_ vs. the other ones are depicted in Table 1; only BXL-01-0029 displayed an IC_50_ value statistically significant vs. all the other tested molecules, but elocalcitol.

### CXCL10 Secretion by Hfsmc was Specifically Induced by TNFα and IFNγ Synergy and Impaired by the Inhibition of TNFα-related Intracellular Pathways

To verify the specificity of synergy between IFNγ and TNFα onto CXCL10 protein secretion in Hfsmc, we incubated the cells with IFNγ (1000 U/ml) or TNFα (10 ng/ml) alone or combined; either IFNγ or TNFα were combined with IL-12 (50 ng/ml), IL-18 (50 ng/ml), IL-2 (25 U/ml) and IL-4 (20 ng/ml). The effect of each single cytokine was tested.

**Figure 2:**
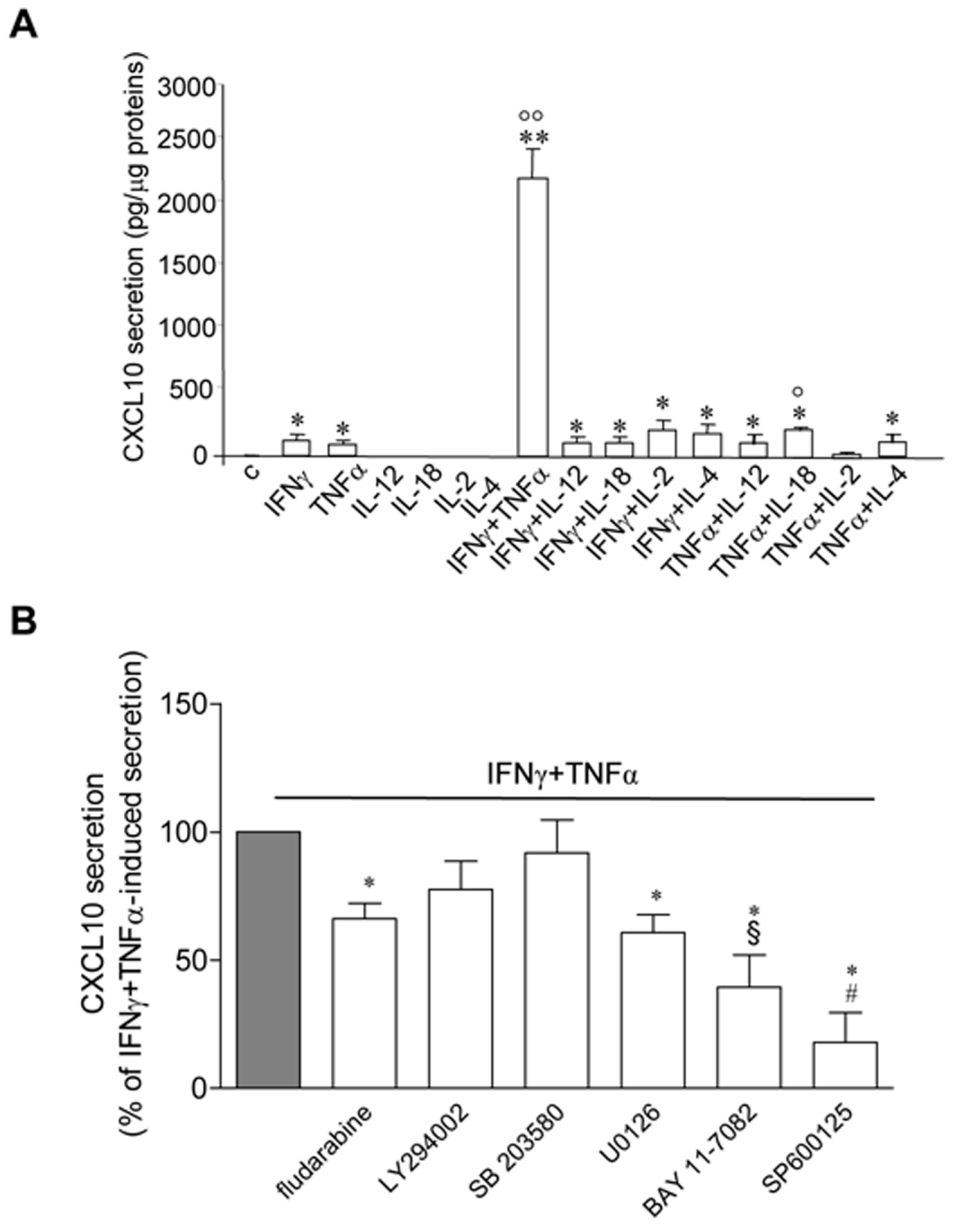
Evaluation of IFNγ+TNFα synergy and intracellular pathways involved in CXCL10 secretion by Hfsmc. A, CXCL10 secretion, virtually absent in control, was significantly increased by 24γ (1000 U/ml) and TNFα (10 ng/ml) and enhanced at the highest level only by their combination; *P<0.05 or **P<0.01 vs. control; °°P<0.01 vs. each other cytokines, single or combined. IL-12 (50 ng/ml), IL-18 (50 ng/ml), IL-2 (25 U/ml) and IL-4 (20 ng/ml) had effect onto neither IFNγ- nor TNFα-induced CXCL10 secretion, except for IL-18 combined with TNFα (°P<0.05 vs. TNFα-induced secretion). Data are expressed as pg/µg protein, mean±S.E. Results are obtained from six experiments with different cell preparations. B, IFNγ+TNFα-induced CXCL10 secretion was significantly reduced by the blockade of different intracellular pathways with the specific inhibitors: fludarabine (Stat-1) 66.08±6.05%; LY294002 (PI3K) 77.6±10.97%; SB 203580 (p38 MAPK) 91.70±12.86%; U0126 (ERK1/2) 60.70±6.88%; BAY 11–7082 (NF-kB) 39.40±12.71%; SP600125 (JNK) 17.84±10.10%. *P<0.05 vs. IFNγ+TNFα-induced secretion, taken as 100%; ^#^P<0.05 vs. all other treatments except for BAY 11–7082; ^§^P<0.05 vs. SB 203580. Results (mean±S.E.) are expressed as % of IFNγ+TNFα-induced CXCL10 secretion (taken as 100%). Data are obtained from five experiments with different cell preparations.

Only IFNγ and TNFα combination (Figure 2A) elicited the highest CXCL10 secretion (2207.84±239.80 pg/µg proteins, P<0.01 vs. control and vs. each other cytokine alone or combined). With the exception of IFNγ and TNFα (122.37±36.73 and 90.07±23.16 pg/µg proteins, respectively, P<0.01 vs. control), none of the single tested cytokine induced CXCL10 secretion by Hfsmc. TNFα-induced CXCL10 secretion was enhanced by IL-18 (214.96±32.90 pg/µg proteins, P<0.05 vs. TNFα-treated cells).

We next investigated the intracellular pathways involved in CXCL10 protein secretion induced by IFNγ+TNFα in Hfsmc.

Cells were incubated in presence of IFNγ (1000 U/ml)+TNFα (10 ng/ml) with and without the selective inhibitors of Stat-1 (fludarabine), PI3K (LY294002), p38 MAPK (SB 203580), ERK1/2 (U0126), NF-kB (BAY 11–7082), and JNK (SP600125). All the inhibitors, with the exception of LY294002 and SB 203580, reduced cytokine-induced CXCL10 secretion (P<0.05 vs. IFNγ+TNFα-induced secretion, taken as 100%) (Figure 2B). In particular, CXCL10 secretion was mostly reduced by JNK and NF-kB blockage (more than 80% and 60%, respectively; JNK inhibition: P<0.05 vs. all other treatments except for BAY 11–7082; NF-kB inhibition: P<0.05 vs. SB 203580).

### BXL-01-0029 Inhibited Signal Pathways Downstream of TNFα and TNFα-induced CXCL10 Secretion in Hfsmc

**Figure 3:**
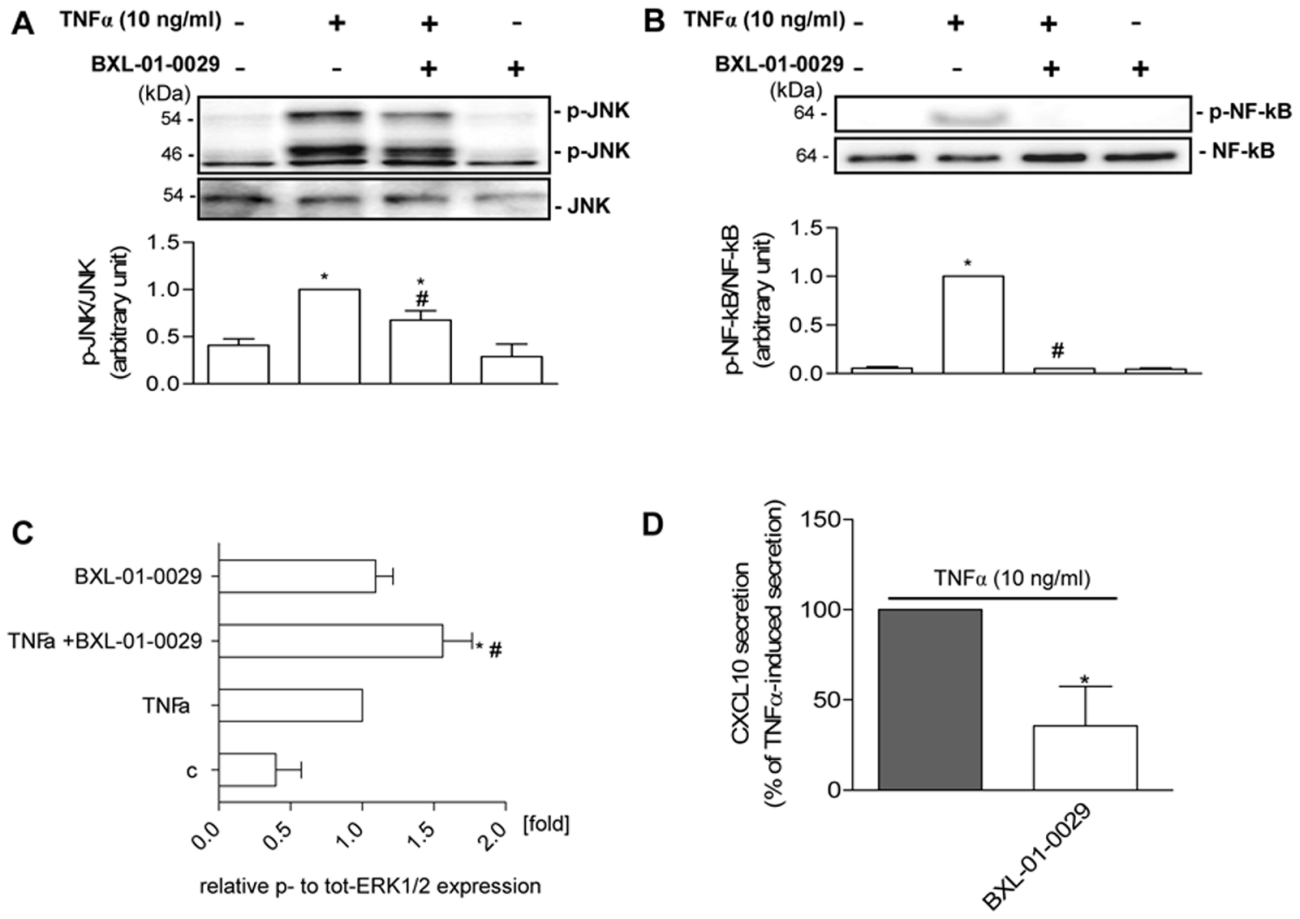
Effect of BXL-01-0029 onto TNFα-treated Hfsmc. A, B, Western blot analysis was performed to assess JNK (A) and NF-kB (B) activation after 12 min stimulation with TNFα (10 ng/ml), with or without BXL-01-0029 (10^−8^ M). TNFα induced JNK and NF-kB phosphorylation; BXL-01-0029 impaired JNK activation and prevented NF-kB phosphorylation (upper A and B); total JNK and NF-kB were used as loading control (middle A and B). Lower A and B report densitometric analysis (*P<0.05 vs. control, ^#^P<0.05 vs. TNFα-treated cells) and results are expressed as ratio phosphorylated/total protein arbitrary units vs. TNFα-treated cells, taken as 1 (mean±S.E.). C, Bio-Plex protein analysis assessed ERK1/2 activation after 12 min stimulation with TNFα (10 ng/ml), with or without BXL-01-0029 (10^−8^ M). TNFα and BXL-01-0029 induced ERK1/2 phosphorylation (*P<0.05 vs. control); their combination enhanced this effect (^#^P<0.05 vs. TNFα-treated cells). Results are expressed as fold of relative p- to tot-ERK1/2 expression vs. TNFα-treated cells, taken as 1 (mean±S.E.). Data were obtained from two experiments with different cell preparations. D, TNFα-induced CXCL10 secretion, taken as 100%, was significantly reduced by BXL-01-0029 (10^−8^ M) after 24 h incubation; *P<0.05 vs. TNFα-induced secretion. Results (mean±S.E.) are derived from five experiments, using distinct cell preparations.

We next found that BXL-01-0029 in Hfsmc was able to impair TNFα signaling by interfering with JNK, NF-kB and ERK1/2 phosphorylation, as shown by protein analysis with western blot (Figures 3A, 3B) or Bio-Plex® suspension array system (Figure 3C).

In particular, the treatment of Hfsmc with TNFα significantly increased the phosphorylation of JNK, NF-kB and ERK1/2 vs. control (Figures 3A, 3B and 3C, P<0.05); the simultaneous incubation with BXL-01-0029 significantly reduced TNFα-induced activation of JNK and virtually prevented NF-kB phosphorylation (Figures 3A and 3B, upper), as confirmed by the densitometric analysis (Figures 3A and 3B lower, P<0.05 vs. TNFα-treatment, taken as 1); VDR agonist alone exerted no effect. Total JNK and NF-kB were used for loading controls (Figures 3A and 3B, middle).

BXL-01-0029 induced ERK1/2 phosphorylation similarly to TNFα (taken as 1, P<0.05 vs. control, Figure 3C) and exerted an additive effect when combined with TNFα, as revealed by Bio-Plex analysis (P<0.05 vs. TNFα-treatment). BXL-01-0029 significantly decreased TNFα-induced CXCL10 protein secretion by Hfsmc (Figure 3D).

### BXL-01-0029 did not Affect Signal Pathway Downstream of IFNγ and IFNγ-induced CXCL10 Secretion in Hfsmc

**Figure 4:**
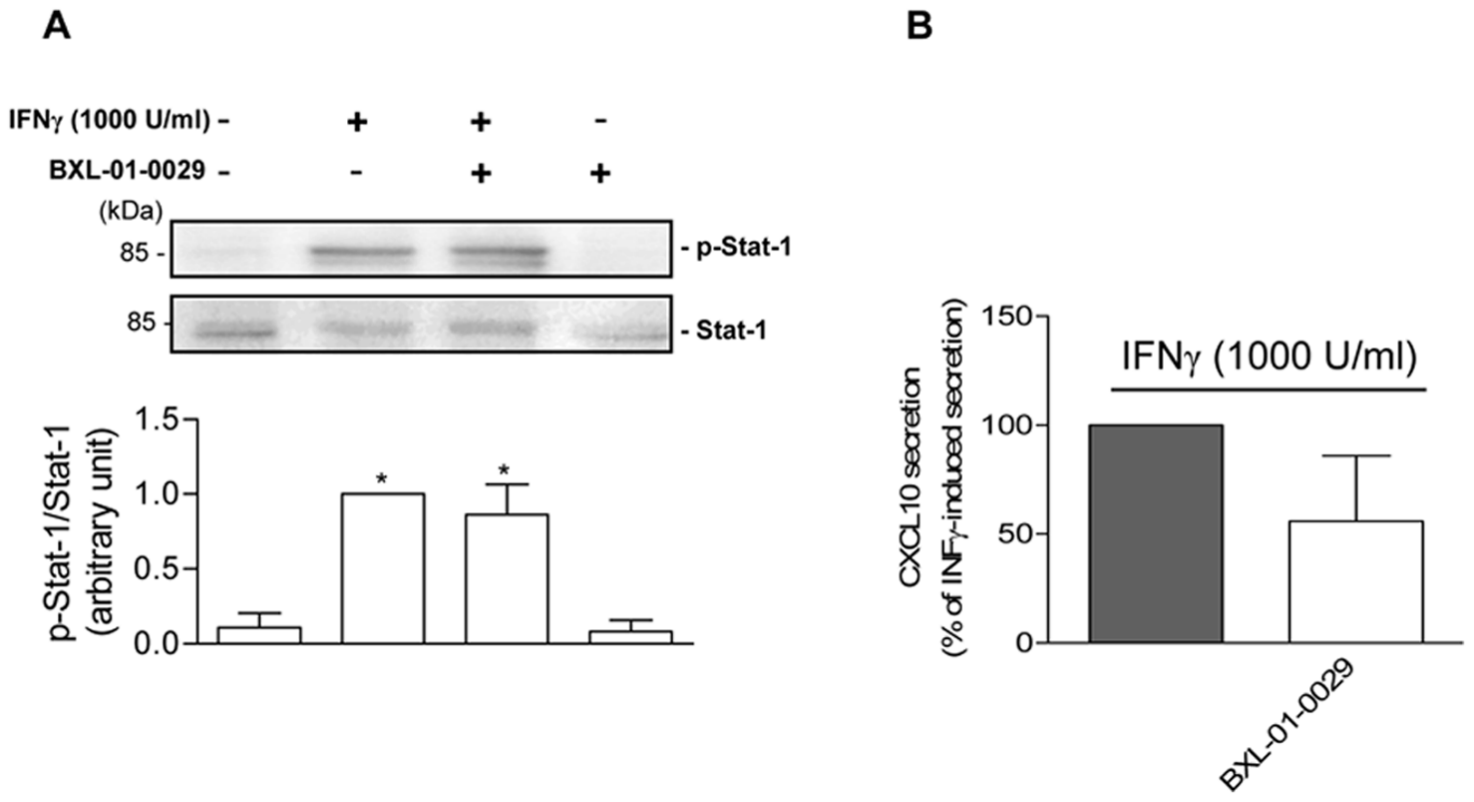
Effect of BXL-01-0029 onto IFNγ-treated Hfsmc. A, Western blot analysis to assess Stat-1 activation after 12 min stimulation with IFNγ (1000 U/ml), in presence or absence of BXL-01-0029 (10^−8^ M) showed that IFNγ increased Stat-1 phosphorylation, whereas BXL-01-0029 did not exert any effect (upper A); total Stat-1 was used as loading control (middle A). The densitometric analysis is reported in lower A (*P<0.05 vs. control) and results are expressed as ratio phosporylated/total protein arbitrary units vs. IFNγ-treated cells, taken as 1 (mean±S.E.). B, Cell incubation for 24 h with BXL-01-0029 (10^−8^ M) did not affect IFNγ-induced CXCL10 secretion, taken as 100%. Results (mean±S.E.) are derived from five experiments, using distinct cell preparations.

BXL-01-0029 did not exert a significant effect onto Stat-1 phosphorylation induced by IFNγ, taken as 1, (Figure 4, upper), as confirmed by densitometric analysis (Figure 4 lower, P<0.05 vs. control); total Stat-1 used as loading control is depicted (Figure 4A, middle). IFNγ-induced CXCL10 secretion (taken as 100%, Figure 4B) was unaffected by BXL-01-0029.

### BXL-01-0029 Counteracted Cytokine-induced Class II HLA Expression in Hfsmc

**Figure 5:**
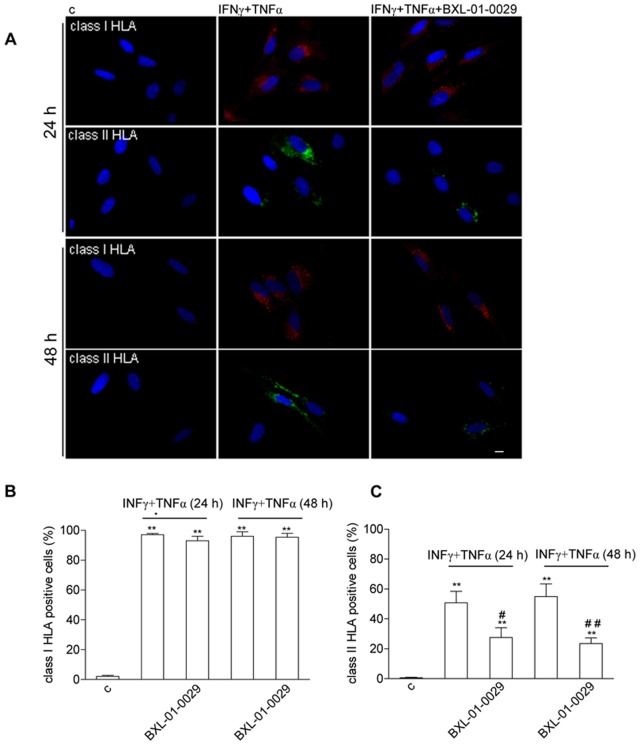
Effect of BXL-01-0029 on IFNγ+TNFα-induced class I and class II HLA in Hfsmc. A, Immunocytochemistry revealed no constitutive expression of class I and class II HLA in control Hfsmc (left A); positive staining for both antigens was observed after 24–48 h incubation with IFNγ+TNFα (middle A); BXL-01-0029 significantly counteracted cytokine-induced class II HLA expression at both time points, with no effect onto class I HLA (right A). Pictures are representative. B, C, Cells were scored as either positive or negative for class I and class II HLA, respectively. Stained cells for each antigen were virtually absent in control cells (class I HLA: 2.3±0.19% and 0.5±0.3% for class I and II HLA, respectively). Stimulation with IFNγ+TNFα induced virtually all Hfsmc to express class I HLA (98.8±0.8% at 24 h; 99.1±0.9 at 48 h, **P<0.01 vs. control) and about half of the cells to express class II HLA (50.75±6.86% at 24 h; 54.96±2.21% at 48 h, **P<0.01 vs. control). The simultaneous incubation with BXL-01-0029 significantly decreased the number of class II HLA-stained cells after 24 h (27.51±5.81%, ^#^P<0.05 vs. cytokine-treated cells) and 48 h (23.54±3.31%, ^##^P<0.01 vs. cytokine-treated cells). Results are obtained with four different cell preparations and expressed as the percentage of positive cells over the total (mean±S.E.).

Class I and class II HLA expression, virtually absent in control Hfsmc (Figure 5A, left), significantly increased after the treatment with IFNγ (1000 U/ml)+TNFα (10 ng/mL) for 24–48 h: almost 100% increase of class I at both time points; more than 50% increase of class II HLA, at 24 and 48 h, respectively (Figure 5A, middle and Figure 5B, 5C). The simultaneous addition of BXL-01-0029 (10^−8 ^M) did not affect the number of cytokine-induced class I HLA positive cells, at each time point (Figure 5, right and Figure 5B). Conversely, after 24–48 h, the VDR agonist significantly decreased the number of class II HLA positive cells vs. cytokine-treated cells (at 24 h: 27.51±5.81% with BXL-01-0029 vs. 50.75±6.86% with IFNγ+TNFα, P<0.05; at 48 h: 23.54±3.31% with BXL-01-0029 vs. 54.96±2.21% with IFNγ+TNFα, P<0.01; Figure 5, right and Figure 5C). Figures are representative. No effects were observed in cells treated with BXL-01-0029 alone or processed for method control (not shown).

### CXCL10 Serum Level was the Highest in IM Patients at the Time of Diagnosis

**Figure 6:**
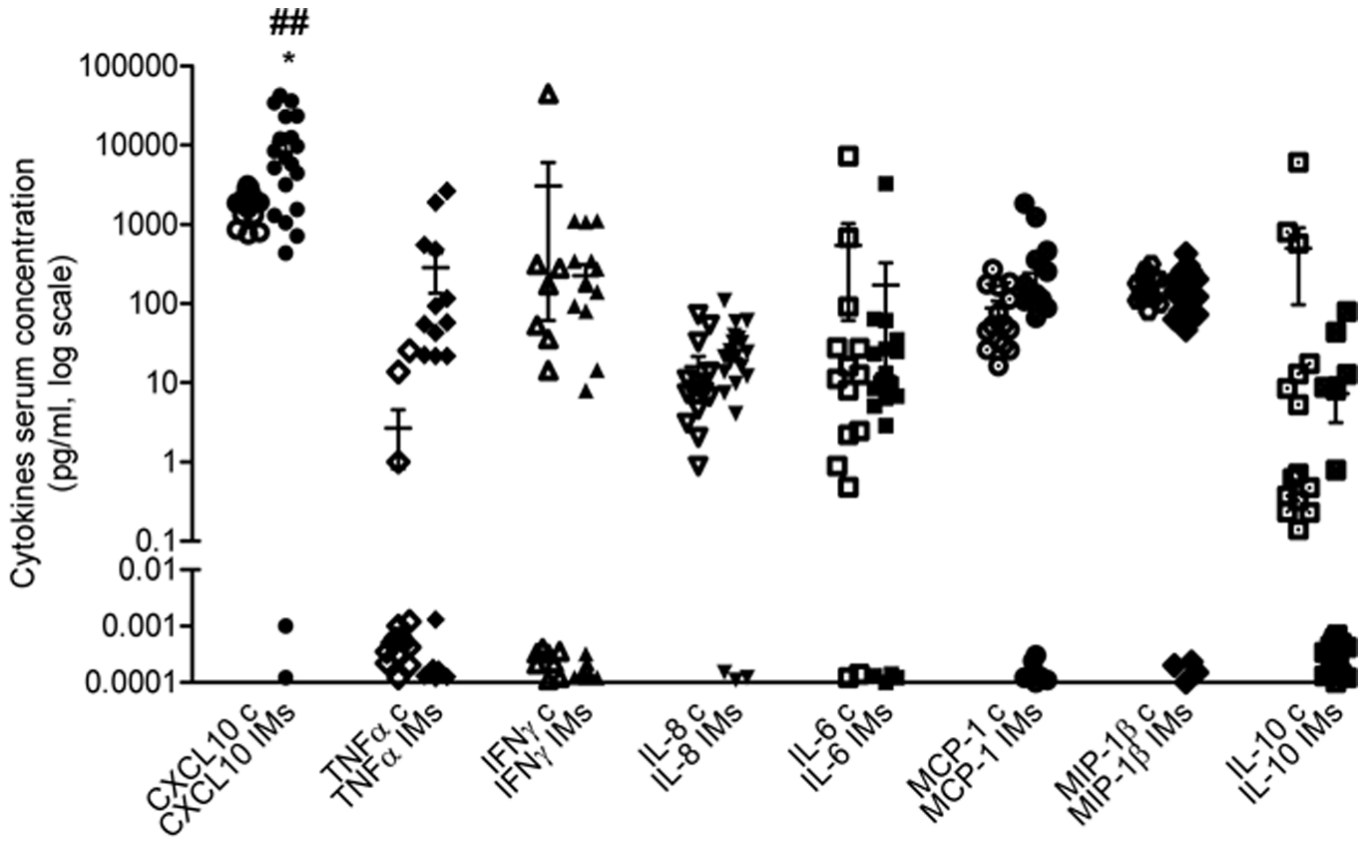
Circulating level of different cytokines in IM patients. Analysis with Bio-Plex suspension array system of CXCL10, TNFα, IFNγ, IL-8, IL-6, MCP-1, MIP-1β and IL-10 in IM patients and matched control subjects. CXCL10 circulating level was significantly higher in IM patients vs. controls and the highest out of the other tested analytes; *P<0.05 vs. control, ^##^P<0.01 vs. each other tested analyte.

In subjects diagnosed with IMs before therapy initiation CXCL10 serum level was the highest out of the other measured cytokines TNFα, IFNγ, IL-8, IL-6, MCP-1, MIP-1β (Th1 type) and IL-10 (Th2 type) (P<0.01, Figure 6), and the only one significantly different from matched healthy controls (P<0.05).

## Discussion

This study highlights for the first time that a VDR agonist counteracted cytokine-induced CXCL10 secretion by human skeletal muscle cells throughout TNFα pathway deactivation. The IC_50_ values documented that BXL-01-0029 targeted human skeletal muscle cells by inhibiting TNFα+IFNγ-induced CXCL10 secretion with the highest potency as compared to the other tested immunosuppressants. This effect might be due, at least in part, to the impairment of NF-kB and JNK intracellular pathways, both downstream of TNFα [33], [34]; remarkably, BXL-01-0029 seems to prevent NF-kB activation, as shown by the virtual absence of its phosphorylation in presence of the VDR agonist. This latter observation is in line with the effect previously reported in human cardiomyocytes [21]; indeed, NF-kB and TNFα-related pathways are well known vitamin D targets [35]. Accordingly, CXCL10 secretion induced by TNFα, alone or combined with IFNγ, was significantly decreased by BXL-01-0029. Based on the result observed on ERK1/2 signaling pathway, also downstream of TNFα, we could speculate that BXL-01-0029 likely affects VDR activity in Hfsmc; indeed, it is known that VDR ligands interfere with ERK signaling by rapid mechanism. While ERK pathway activation is associated with the significant increases in pro-inflammatory cytokine transcription [36] during muscle inflammation, the effect of ERK activation on VDR transcriptional activity (i.e., enhancement or attenuation) is cell specific and depends on the type of the retinoid X receptor (RXR) engaged as heterodimer partner, such as α, β and γ isoforms, all expressed by human skeletal muscle [37].

As from our data in Hfsmc, BXL-01-0029 seems to selectively target TNFα signaling pathway without interference onto IFNγ, as shown by no significant changes either in Stat-1 phosphorylation or in IFNγ-induced CXCL10 secretion. This result is quite different from the inhibition of IFNγ-induced Stat-1 phosphorylation and activation previously observed in human thyrocytes and cardiomyocytes treated with BXL-01-0029 or elocacitol, respectively [20], [21]; hence, we could hypothesize a different/specific effect of VDR agonists depending also on target cell types.

Herein, we documented that the significant CXCL10 protein secretion by Hfsmc - virtually absent in basal condition – as observed under inflammatory challenge occurred in association to NF-kB, JNK or ERK intracellular path activation, whose specific blockage significantly reduced chemokine secretion; class I and II HLA expression was consistently increased as well. The latter effect is in line with data from other groups showing an upregulation of class I and II HLA both *in vivo* in IM and *in vitro* upon pro-inflammatory cytokine stimulation [38]–[42]. At variance with previous studies in human myoblasts, constitutive HLA I was not detectable in Hfsmc: this may be due to the reduced/different immunogenicity of fetal cells [43]–[45]. However, Hfsmc - capable to respond to an allogenic *in vitro* stimulation, as previously shown [10] - have now been documented to be equipped with the major constituents necessary for antigen processing. In this view, the compliance of Hfsmc as an optimal *in vitro* tool for basic research in human muscle inflammation has been confirmed. Differently from class I HLA, whose induction by proinflammatory stimuli did not change with VDR agonist, cytokine-induced class II HLA expression in Hfsmc seems to be counteracted by BXL-01-0029. This result is quite intriguing since class II HLA class molecules lead to the immunogenic presentation of autoantigents to CD4+T cells [39]. Remarkably, cytokines have been shown to contribute to pathogenesis of IM by upregulating HLA [46] and TNFα, in particular, has been recently hypothesized as a key mediator and potential therapeutic target in T cell mediated IM [42]. The association between HLA, TNFα polymorphisms and genetic risk for the pathogenesis of myositis has been reported in subjects with polimyositis, dematomyosistis or myositis overlapping with other connective diseases [47]. As from studies in IM patients and murine cells, TNFα-mediated pathway seems to mediate the modification of the alternative splicing factor ASF/SF2, which is known to have a major role in inflammation and autoimmune muscle diseases [48]. TNFα, quite differently from IFNγ - rapidly degraded and, therefore, not always easily detectable - is highly expressed in tissues and blood of IM patients [5], [49] and it seems the pivotal cytokine in promoting muscular inflammation at cellular level [50], [51]. The importance of TNFα in myositis development has been recently confirmed and highlighted in an animal model [52]. Accordingly, we have previously shown that the system TNFα-TNFα receptor (TNFαR) type II is likely the critical one in promoting muscular inflammation at cellular level in Hfsmc, throughout the synergy with IFNγ. As documented by the combination with some other Th1 type cytokines, known to be engaged in skeletal muscle inflammation, such as IL-12, IL-18, IL-2 [53], [54], or with IL-4 (Th2 type), there is a specific synergistic effect only in presence of TNFα+IFNγ; such a specificity is associated to a significant up-regulation of TNFα type II receptor [10], the subtype mainly engaged in immune response regulation, as we previously reported [55], [56].

In clinics, pharmacological blockade of TNFα activity – i.e., by the neutralizing antibody infliximab or the soluble TNFαR Etanercept – used to reduce symptoms of rheumatoid arthritis or Crohn’s disease, has been since few years ago extended also to IMs, although some caution has been recommended [18], [57]–[59]. A screening for detecting potentially valuable inhibitors of NF-κB, in order to block TNFα activity and identify non toxic optimal therapies for the inflammatory myopathies, has been repoted in a murine cell line [60].

So far, while our study is in keeping with previous *in vivo* and *in vitro* observations, it provides for the first time *in vitro* evidence for human skeletal muscle cell contribution to immune/inflammatory processes through TNFα pathway, and, more importantly, it highlights potential target(s) at muscle cell level. In our opinion, the use of human cellular system is a key point in basic research, in order to avoid any species-specificity bias.

Our data indirectly confirm and sustain the critical role of TNFα in human IM pathogenesis, providing new insight(s) at molecular level: as from previous [10] and present results, CXCL10 production and its main triggering path, the intracellular cascade downstream of TNFα, have emerged as potential new therapeutic targets in muscular inflammation. Muscular cells, although being not the main source *in vivo*, can produce CXCL10 during inflammatory conditions, as shown by immunohistochemical studies in skeletal muscle fibers of patients with juvenile dermatomyositis [15]. CXCL10 not only mediates leukocyte recruitment and infiltration at the site of inflammation during the early stages of auto- or alloimmune response, but, and even more important, likely triggers the reaction following the antigenic challenge [17]; in this light, we hypothesize that pharmacological targeting local production of CXCL10 by VDR agonist could be a novel helpful therapeutic approach to inflammatory disorders of the muscle. Remarkably, circulating CXCL10 level was the highest as compared to each other tested Th1 cytokine level in sera of subjects at time of diagnosis with IMs; furthermore, only CXCL10 was significantly higher in patients than in matched controls. However, we’d like to underline some difference in TNFα serum level between the two groups of subjects, although not statistically significant, probably due to sample variability. Of note, some of our *in vivo* results obtained at the time of diagnosis are in line with a previous study on some circulating Th1 type cytokines in subjects with different types of IMs, in active phase of the diseases [49]. Albeit many of the immunopathogenic processes behind IMs remain poorly understood, is generally accepted that cytokines and chemokines, including IFNγ, TNFα and CXCL10, are pivotal players and may be defined as “disease-perpetuating” molecules; correlational clustering is able to discriminate between, and, hence, sub-classify patients with IMs [1], [49]. In addition, cytokine profile during treatment - before and after MeP, CsA, MTX or intravenous Ig - permitted to identify some key cytokines clearly reflecting the disease improvement, thus to be considered as excellent candidates for therapeutic efficacy indicators [49].

Current therapeutic approaches for IMs target mainly immune cells, as mentioned above, and are based mostly on empirical evidence; the absence of internationally validated evaluation criteria to conduct randomized controlled trials - due both to low prevalence and heterogeneity of IMs - represents a major concern in clinics [18]; the inadequate response and the intolerance to the therapy, often encountered by IM patients, predict a poor outcome. Thus, new agents designed to target specific different component(s) to the immune response other than immune cells, such as muscular cells, would be high attractive. The finding that in human skeletal muscle cells BXL-01-0029 is the most potent inhibitor of CXCL10 secretion, by specifically targeting the intracellular signal pathway downstream of TNFα, might be quite intriguing in view of new potential pharmacological tool(s) for the treatment of IMs, as above reported. Thus far, based on the rapid actions as observed onto intracellular path activation, we could hypothesized a non genomic effect of VDR analogue; however, alternative mechanism(s) of action, i.e., involving effects on other intracellular pathway(s) or cytokine receptor(s) cannot be excluded. It is also noteworthy that both BXL-01-0029 and elocalcitol have been documented to significantly decrease CXCL10 secretion by CD4^+^T cells without any cytotoxic effect onto cell viability, at variance with some other immunosuppressants [9],[21]. We want to remark that we selected drug concentrations on the basis of their near-therapeutic doses, according to their pharmacokinetics, in order to reflect the serum concentrations of drugs administered to patients.

In conclusion, albeit additional studies are mandatory, non or less hypercalcemic VDR agonists might represent optimal candidates to be a potentially more effective and safer therapy, even to combine with the current therapeutic regimens for the treatment of autoimmune muscular diseases, such as IMs.
